# Sensory Loss and Mobility in Aging: Interdisciplinary Perspectives on Challenges and Interventions

**DOI:** 10.1093/geroni/igaf122.075

**Published:** 2025-12-31

**Authors:** Shu Xu, Joshua Ehrlich, Jennifer Deal

## Abstract

This session will discuss sensory loss and mobility in later life from gerontological, public health, and intervention perspectives. Sensory loss is highly prevalent among older adults, with over half experiencing hearing loss and more than a quarter living with visual impairment in the U.S. As the population ages, the number of older adults with sensory loss are expected to increase accordingly. Sensory health is essential for maintaining mobility, as sensory loss is linked to poor postural balance, fall-related issues, walking difficulties, reduced participation in daily activities. However, the association between sensory loss and various forms of mobility remains underexplored. Environmental factors and the potential mechanisms underlying these associations are under-investigated. This session will investigate associations between objectively measured hearing, vision, dual sensory loss and outdoor mobility limitations, along with environmental factors, such as public transit, that may help explain these associations. We will examine the longitudinal association between different types of visual function and physical performance using a nationally representative sample of U.S. older adults. We will also describe characteristics of communities with high rates of hearing difficulties and falls using community-level data. Whether treatment of sensory loss is an effective intervention for improving mobility is unknown. This session will conclude by presenting results of a randomized controlled trial investigating the effects of hearing care on daily functioning in older adults with hearing loss. Bringing together researchers from multiple disciplines, this symposium will advance understanding of sensory loss and mobility in aging, with implications for research, policy, and practice.

